# mRNA Expression in Papillary and Anaplastic Thyroid Carcinoma: Molecular Anatomy of a Killing Switch

**DOI:** 10.1371/journal.pone.0037807

**Published:** 2012-10-24

**Authors:** Aline Hébrant, Geneviève Dom, Michael Dewaele, Guy Andry, Christophe Trésallet, Emmanuelle Leteurtre, Jacques E. Dumont, Carine Maenhaut

**Affiliations:** 1 Institute of Interdisciplinary Research, Free University of Brussels, Brussels, Belgium; 2 Laboratory for Molecular Cancer Biology, Flanders Institute for Biotechnology, LEUVEN, Belgium; 3 Institut Jules Bordet, Bruxelles, Belgium; 4 Hôpital Pitié Salpêtrière, Paris, France; 5 Université de Lille 2, Faculté de Médecine, Lille, France; 6 Centre Hospitalier Régional Universitaire de Lille, Institut de Pathologie, Lille, France; 7 Walloon Excellence in Lifesciences and Biotechnology, Free University of Brussels, Brussels, Belgium; Consiglio Nazionale delle Ricerche (CNR), Italy

## Abstract

Anaplastic thyroid carcinoma (ATC) is the most lethal form of thyroid neoplasia and represents the end stage of thyroid tumor progression. No effective treatment exists so far. ATC frequently derive from papillary thyroid carcinomas (PTC), which have a good prognosis. In this study, we analyzed the mRNA expression profiles of 59 thyroid tumors (11 ATC and 48 PTC) by microarrays. ATC and PTC showed largely overlapping mRNA expression profiles with most genes regulated in all ATC being also regulated in several PTC. 43% of the probes regulated in all the PTC are similarly regulated in all ATC. Many genes modulations observed in PTC are amplified in ATC. This illustrates the fact that ATC mostly derived from PTC. A molecular signature of aggressiveness composed of 9 genes clearly separates the two tumors. Moreover, this study demonstrates gene regulations corresponding to the ATC or PTC phenotypes like inflammatory reaction, epithelial to mesenchymal transition (EMT) and invasion, high proliferation rate, dedifferentiation, calcification and fibrosis processes, high glucose metabolism and glycolysis, lactate generation and chemoresistance. The main qualitative differences between the two tumor types bear on the much stronger EMT, dedifferentiation and glycolytic phenotypes showed by the ATC.

## Introduction

Thyroid tumors are divided into encapsulated benign tumors (autonomous and follicular adenomas) and carcinomas. These carcinomas are themselves subdivided into differentiated carcinomas (follicular carcinomas (FTC) or papillary carcinomas (PTC)) which may evolve into the very aggressive and dedifferentiated anaplastic carcinomas (ATC) [1]. ATC share genetic alterations with FTC and PTC, namely, BRAF, RAS, PTEN and PI3KCA mutations or gene amplifications [1].

Despite its low frequency (<5% of all thyroid carcinomas), ATC is responsible for more than half of thyroid carcinoma deaths, with a mean survival of 6 months after diagnosis [2]. Benefits obtained from chemotherapy and radiation therapy remain only marginal and there are no alternative treatments yet [3]; [4]. New approaches are therefore needed.

mRNA expression analysis based on microarray technology has been largely used to characterize human cancers. This approach allows the identification of genes important in the tumorigenesis process, and the definition of diagnosis and prognosis signatures.

Until now, only a limited number of ATC have been investigated for mRNA expression with incomplete and sometimes not very sensitive microarray sets [1]; [5]; [6]. Moreover, no systematic comparison between PTC and ATC has been made previously. This is the first exhaustive study of gene expression comparing normal thyroid tissues, PTC and ATC, using full genome microarrays.

To identify the molecular mechanisms involved in tumor evolution, we analyzed the mRNA expression profiles of 59 thyroid tumors (11 ATC and 48 PTC) using the Affymetrix microarray technology and real-time qRT-PCR and the mutational status of 11 ATC.

The analysis of the genes regulated in ATC revealed several very interesting known and unknown features: a strong similarity with PTC, a signature of 9 genes discriminating ATC and PTC which may be related to clinical prognosis, and biological signatures which suggest new therapeutic approaches. The study defines the molecular phenotypes corresponding to the qualitatively described pathological features of these cancers.

## Materials and Methods

### Tissue Samples

16 ATC and 53 PTC were obtained from different hospitals: Regional Reference Cancer Center of Lille (Lille, France), Pitié-Salpêtrière (Paris, France), Jules Bordet Institute (Brussels, Belgium), Cliniques Universitaires Saint-Luc (Brussels, Belgium), Katholieke Universiteit Leuven (Leuven, Belgium) and from the Chernobyl Tissue Bank (www.chernobyltissuebank.com). Eleven ATC and 48 PTC tumors (classical variants) destinated for microarray hybridizations were compared to a reference pool of 23 normal, non-neoplastic thyroid tissues from the contra-lateral lobe with respect to the thyroid carcinomas. The remaining 5 ATC and 5 PTC were used as independent samples for validation. Tissues were immediately dissected, placed on ice, snap-frozen in liquid nitrogen and stored at −80°C until RNA processing. Protocols have been approved by the ethics committees of the Institutions.

### RNA Purification

Total RNA was extracted from thyroid tissues using Trizol reagent (Invitrogen), followed by purification on RNeasy columns (Qiagen). The RNA concentration was spectrophotometrically quantified, and its integrity was verified using an automated gel electrophoresis system (Experion, Biorad).

### Mutation Screening

In order to determine the mutational status for TP53, BRAF, H-RAS, N-RAS, K-RAS, PI3KCA and β-catenin in the 11 ATC samples, the sequences containing the most frequent mutations were amplified by PCR using appropriate primer pairs (primer sequences and PCR conditions provided in Table S1). PCR products were sequenced by Big Dye Terminator cycle sequencing on an automated ABI Prism 3100 sequencer (Applied Biosystems, Foster City, USA).

### Microarray Hybridization

Two µg of total RNA from 11 ATC and 48 PTC were engaged for cDNA synthesis. Labeled cRNA was synthesized, purified and hybridized on Affymetrix HU 133 Plus 2.0 arrays, following the Affymetrix Protocol.

### Data Acquisition and Bioinformatic Analyses

#### Data acquisition, background correction and normalization

CEL file data were normalized by GCRMA. For each spot, data were expressed as the log_2_ ratio of fluorescence intensities of the tumor tissue and the reference normal tissues pool. All gene expression data are released on GEO under the accession number GSE33630.

#### Selection of commonly deregulated genes

The subset of probes that varied by at least 2-fold compared to the normal pool (ratios tumor/normal) in all the 11 ATC samples (without any opposite regulation) was selected and called the ATC list. Similarly, those that varied by at least 2-fold compared to the normal pool in all the 48 PTC samples (without any opposite regulation) were selected and called the PTC list.

#### Search for a signature of aggressiveness

Probes which were upregulated more than 1.5-fold in all the ATC and downregulated or not modulated in PTC, and probes which were downregulated more than 1.5-fold in all the ATC and upregulated or not modulated in PTC were selected for a potential signature of aggressiveness of ATC versus PTC. Similarly, probes which were upregulated more than 1.5-fold in all the PTC and downregulated or not modulated in ATC and probes which were downregulated more than 1.5-fold in all the PTC and upregulated or not modulated in ATC were also selected for this aggressiveness signature.

#### Nonsupervised analyses

Nonsupervised analyses were performed on the basis of between-sample correlation distances. Multidimensional scaling (MDS, as implemented by R isoMDS function) was performed considering all the probes present on the microarray with the 11 ATC and the 48 PTC samples.

### Validation of Microarray Results by Quantitative Real-time RT-PCR (qRT-PCR)

A number of modulated genes on the microarray slides were validated using qRT-PCR (SYBR Green, Eurogentec, Liege, Belgium). The following mRNA expressions were evaluated using, when possible, transexonic primers, designed with Primer Express software (Applied Biosystems): NELL2, SPINT2, MARVELD2, DUOXA1, RPH3AL, TBX3, PCYOX1, c5orf41, PKP4 (primers sequences provided in Table S2). qRT-PCR were performed in triplicate for each gene on 5 new ATC and on 5 new PTC. The data were normalized using TTC1 and NEDD8 mRNA expression [7]; [8].

## Results

### Mutational Status of p53, BRAF, PI3KCA, H-RAS, K-RAS, N-RAS and β-catenin in the 11 ATC

On the 11 ATC, p53 mutation was found in 4 (36%), BRAF mutation in 2 (18%), PI3KCA mutation in 1 (10%). One sample showed both BRAF and p53 mutations (ATC1). No mutation was found for RAS (H-RAS, K-RAS and N-RAS) nor for β-catenin.

### The 11 ATC and the 48 PTC Show Overlapping but Distinct mRNA Expression Profiles

**Figure 1:**
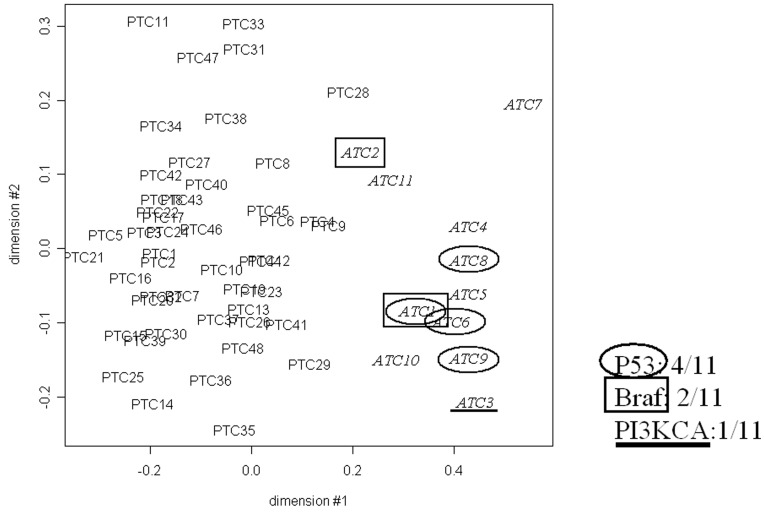
Multidimensional scaling (MDS) of the mRNA expression data from 11 ATC and 48 PTC (all the probes present on the array were considered). The ATC are labelled according to their mutation as depicted in the figure.

Differences in the molecular phenotypes of ATC and PTC can best be demonstrated by a comprehensive microarray analysis of gene expressions in the two types of tissues. Because of the absence of normal tissue counterparts for ATC, gene expression profiles were compared with a common reference pool of 23 normal, non-neoplastic, tissues from the contra-lateral lobe with respect to the thyroid carcinomas. Overall gene expressions from the 11 ATC and the 48 PTC were analyzed using multidimensional scaling (MDS) (Figure 1). The MDS algorithm reduces the n-dimensions space (n: number of probes) into two dimensions while preserving the distances between the samples, and thereby visualizes the similarity relationships between them. MDS showed that the ATC mRNA expression profiles could be distinguished from the PTC ones and that no similar gene expression profiles regarding to their mutational status was observed.

**Figure 2:**
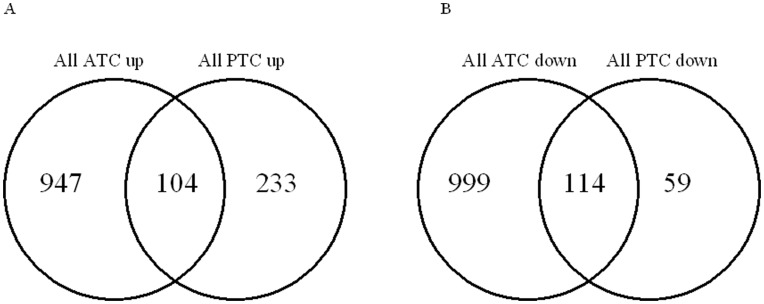
Venn diagrams of the significantly A) upregulated genes in all ATC and all PTC and B) downregulated genes in all ATC and all PTC.

ATC showed 1051 commonly upregulated and 1113 commonly downregulated probes using the criteria of 2 fold described in Material and methods (ATC list (Table S3)). With the same criteria, all PTC presented 337 commonly upregulated and 173 commonly downregulated probes (PTC list (Table S4)). Among the 337 upregulated probes, 104 were regulated in the same way in all the ATC and none in the opposite way. Among the 173 probes downregulated in PTC, 114 were regulated in the same way in all the ATC and none in the opposite way (Figure 2). Thus, 43% of the genes commonly deregulated in PTC were regulated similarly in all the ATC, and no gene was regulated in an opposite way. Generally, the genes whose expression was altered in all the ATC were similarly modulated in many PTC, but the regulations observed were weaker in the latter. Similarly the expression of genes deregulated in PTC was also observed in most ATC.

**Figure 3:**
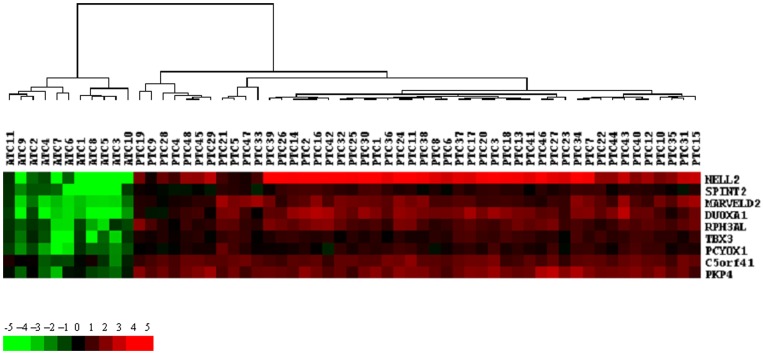
Hierarchical clustering and heat map with the 9 genes composing the signature discriminating ATC and PTC samples.

Using stringent selection criteria (1.5 fold regulation), described in Material and Methods, we identified a minimal signature of 9 genes called signature of aggressiveness (Figure 3) allowing to separate ATC from PTC. We choose 1.5 fold as no gene signature was identified with the 2 fold criteria. All the genes composing this signature were downregulated in ATC and upregulated in PTC and play probably a crucial role in the development of ATC: three of them are related to differentiation (DUOXA1, NELL2 and PCYOX1 which reduces H_2_O_2_ generation), two of them are putative tumor suppressors (SPINT2 and RPH3AL), two others are probably linked to epithelial to mesenchymal transition (EMT) (MARVELD2, a novel tight junction protein and PKP4, thought to be involved in regulating junctional plaque organization and cadherin function), and TBX3 a transcriptional repressor.

### Validation of Microarray Data

The differential expression of several genes in the ATC and PTC lists confirmed the previously published data on ATC by two other groups [5]; [6] (respectively series of 7 and 5 ATC) and on PTC [9]–[12]. Many other gene regulations have not been reported before.

**Figure 4:**
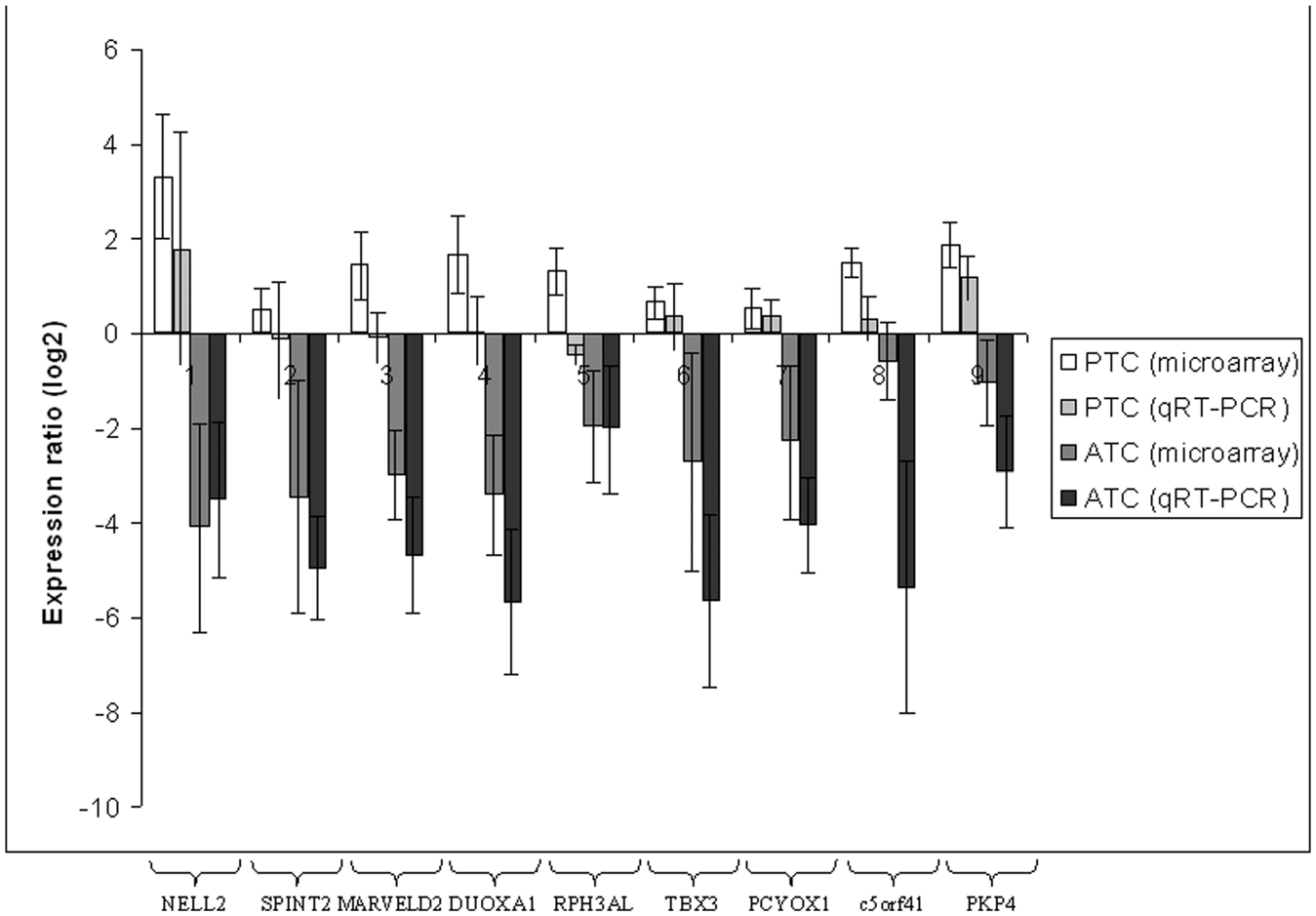
Validation of the 9 genes signature by qRT-PCR on 5 new PTC and 5 new ATC. The microarray expressions is also represented. Log_2_ ratios represent the expression ratios of the genes in the tumors versus a pool of 23 normal thyroid tissues.

To further validate our data, the modulation of the 9 genes composing the signature discriminating ATC from PTC was investigated by real-time qRT-PCR. Experiments were performed on 5 new ATC and on 5 new PTC. Similar modulation patterns were found for the expression of all the genes comparing microarray analysis with qRT-PCR, thus validating the microarray data in independant set of tumors (Figure 4).

### Analysis of the ATC and PTC Gene Lists Suggests a Complete Program Switch from Normal Tissues

First, several immediate early genes (IEG) transcription factors are strongly downregulated in ATC (cJun (Jun), JunD, JunB (9/11), FOS (9/11), FOSB, EGR1, EGR2) attesting a complete program switch between the ATC and the normal tissues. These downregulations were also generally observed in PTC.

Second, there is a switch of gene expression in ATC within functional categories of genes from one set to another: for example, the switch of ion channel genes (e.g. the SLC family with 18 upregulated and 20 downregulated genes), of structural proteins (e.g. the cadherin (CDH family), heatshock proteins (HSP family)), organelle proteins (e.g. ribosomal proteins (RPL family), metabolic enzymes (e.g. aldehyde dehydrogenase (ALDH family)). Most of these switches are common to a great majority of PTC and ATC. The biological meaning of these switches must be analyzed in each case; however collectively they testify to a whole different program of the tumor compared to the normal tissue.

Third, another important level at which changes in protein expression can be regulated is the mRNA stability, as controlled by uridylate rich elements (ARE) in the mRNAs. The proportion of deregulated genes in ATC containing ARE was evaluated by using ARE-mRNA database (ARED:http://rc.kfshrc.edu.sa/ared). The analysis revealed that there are more genes containing ARE in regulated genes than in non regulated genes (26% vs 21%). Moreover, there are more upregulated genes containing ARE than downregulated genes (32% vs 19%). This suggests a role of RNA stability in the ATC phenotype.

### Analysis of the Modulated Genes Using DAVID Software Highlights Specific Pathological Features

**Figure 5:**
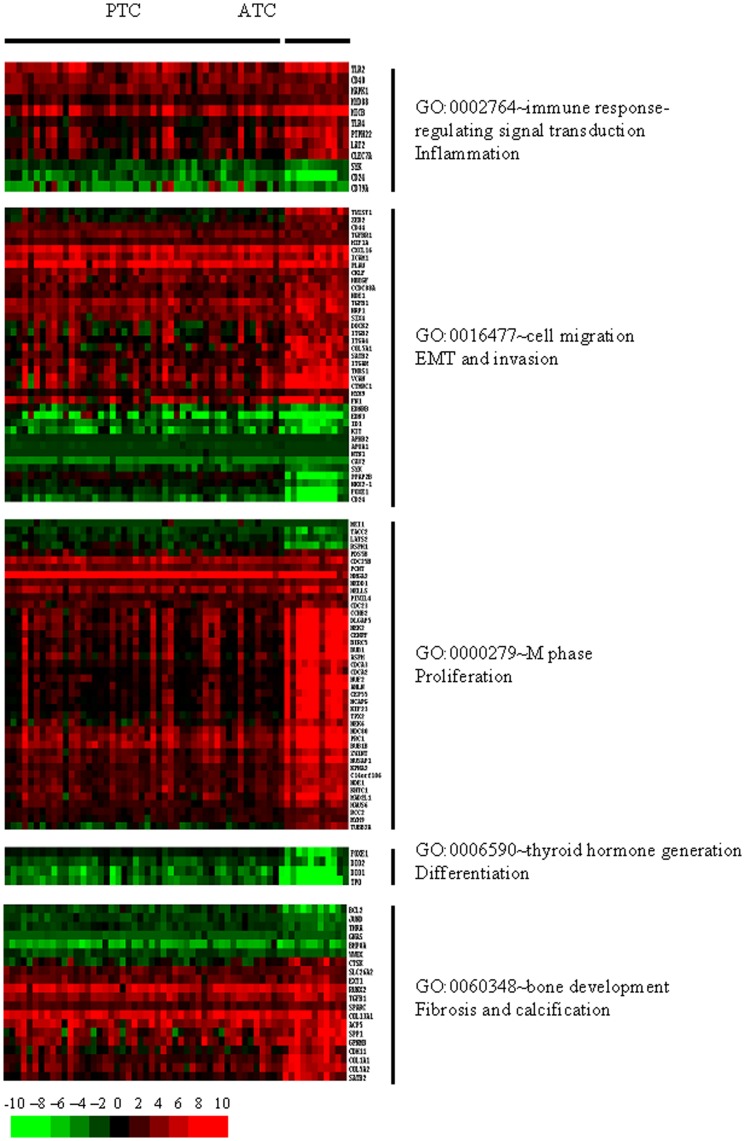
Heat map for genes belonging to some significant Gene Ontology (GO) categories (p-Value <1) corresponding to several biological processes which illustrate the more malignant character of ATC compared to PTC.

The ATC gene list was analyzed by gene ontology (GO) using Database for Annotation, Visualization and Integrated Discovery (DAVID) software [13]; [14]. We observed significant GO categories (p-Value <1) corresponding to several biological processes which illustrate the more malignant character of ATC compared to PTC (Table S5). Among them we selected, for further analysis, altered functions concerning inflammatory reaction, epithelial to mesenchymal transition (EMT) and invasion, proliferation, dedifferentiation, as well as calcification and fibrosis processes (Figure 5).

#### Inflammation

Analysis of Figure 5 shows evidence of a strong inflammatory reaction. The overexpression of CD40 the member of TNF-receptor superfamily, the toll like receptors TLR2 and TLR4, the signal tranducer in the interleukin-1 pathway MYD88, the activator of cytolytic response of natural killer cells MICB, the CLEC7A gene which plays a role in innate immune response are some examples that illustrate this inflammation. The downregulation of SYK and of the B lymphocyte antigen receptors CD24 and CD79A are difficult to explain. The analysis of other genes of the ATC list showed also evidence of a strong inflammatory reaction as supported by the increased expression of other cell surface glycoproteins (CD33, CD44, CD47, CD58, CD68, CD84, CD86, CD97, CD109, CD163, CD209, CD276, CD300A), another toll like receptor (TLR1), interferon γ induced proteins (IFI16, IFI30, IFI44), oligoA synthetases (OAS2, OAS3), tumor necrosis factors (TNFSF4, TNFSF10) and their receptors (TNFRSF19, TNFRSF1B, TNFRSF21), tumor necrosis factor induced proteins (TNFAIP1, TNFAIP3, TNFAIP6), and interleukin receptors of (IL10RA, IL13RA1, IL1RAP). Most of these markers were common to PTC and ATC but were more regulated in ATC than in PTC. Inflammation is observed in many cancers and may have a protumoral role [15].

#### Epithelial to Mesenchymal Transition and Invasion

One of the most striking clinical and pathological feature of ATC is EMT and invasion [16]. EMT is characterized by the reprogramming of epithelial cells in isolated pseudomesenchymal cells progressing independently. These cells are considered as responsible for the first step of metastasis in cancer and show loss of cell-cell contact, remodelling of the cytoskeleton, protease secretion and migration [17].

In the following paragraphs, gene regulations which are not common to all the ATC are indicated into brackets by the fraction of positive tumors (no bracket for those regulated in all ATC).

Biochemically, EMT is characterized by a downregulation of E cadherin (CDH1) (10/11 ATC) and an upregulation of N cadherin (CDH2) (9/11 ATC) and vimentin (VIM). Presumably the decreased expression of CDH16 and the increased expression of CDH11 also reflect this program. Genes of the TGFβ signalling pathway inducing EMT are upregulated (eg. TGFβ1, TGFβ2 (9/11 ATC), TGFβ3 (9/11 ATC), TGFβI and the TGFβR1 receptor) and the expression of transcription factors involved in EMT is also increased (snail (SNAI2), sprouty (SPRY4) (10/11 ATC), zinc finger E-Box binding homeobox (ZEB2 and ZEB1 (8/11 ATC)), twist (TWIST1 and TWIST2 (7/11 ATC))) [17]. Some of the splice isoforms of CD44, upregulated in both ATC and PTC, are essential for EMT [18]. The extracellular matrix proteins and the enzymes involved in matrix metabolism show deregulated expression. In some pathological cases, ATC are diagnosed as fibrosarcomas until the demonstration of some PTC islands [19]. This is also clearly illustrated by the gene expression phenotype: increased expression of mRNA coding for extracellular matrix proteins (collagens (COL5A1), extracellular matrix protein 1 (ECM1), fibronectin (FN1), nidogen (NID1) and laminin (LAMB3)), and for enzymes involved in matrix remodeling (such as lysyl oxidase (LOX), the urokinase plasminogen activator and its receptor (PLAU and PLAUR), matrix metalloproteases (MMP1 and MMP9), and cathepsins (CTSA, CTSC, CTSD, CTSK, CTSS, CTSZ)) [20]–[23], and decreased expression of one receptor which mediates the endocytosis of the LRP1B protein [24]. Some of these expressions have been previously observed [5] and most are similarly regulated in PTC. However, some of them are almost specific markers of the much more aggressive ATC (CDH1, SNAI2, ZEB1, ZEB2, TWIST1 and TWIST2). The specific overexpression of TWIST1 mRNA and protein in ATC has been demonstrated previously [16].

#### Cell prolifération

ATC is a highly proliferative tumor. It is therefore not surprising that gene expression analysis (Figure 5) revealed a general modulation of mRNA coding for proteins involved in proliferation: the upregulation of mRNA coding for proteins involved in cell division and cell cycle checkpoints (CDC25B, CDC23, CDCA2, CENPF, CEP55, BUB1, BUB1B, KIF23, PCNT, CCNB2, NEK2, NEK6, NDE1, KNTC1, MAD2L1, TUBB2A). In addition, negative regulators of proliferation like MEI1, LATS2, stabilized by KIBRA, which stimulates the Hippo pathway itself inhibiting cell proliferation [25] were downregulated. Most of the genes coding for proteins involved in the mitogenic pathways were regulated similarly in PTC and ATC, but more strongly in ATC.

### Differentiation

As expected from their fully dedifferentiated status and as illustrated in Figure 5, when looking at specific thyroid differentiation gene expressions, we observed in ATC a loss of expression of the mRNA encoding three major thyroid determination factors: Pax8, TTF1 (NKX2-1) and TTF2 (FOXE1). In addition, the proteins involved in thyroid specific iodine metabolism, i.e. NIS (SLC5A5) (8/11 ATC), pendrin (SLC26A4) (10/11 ATC), DUOX1 and DUOX2, DUOXA1 and DUOXA2, TPO, Tg (10/11 ATC), DIO1, DIO2 and the sulfotransferase (SULT1C2), and the major protein involved in thyrocyte specific signal transduction, (TSHR) showed decreased expression (http://www.thyroidmanager.org/). The total absence of mRNA of TSHR, Tg and TPO in ATC has already been described [26]. This corresponds well to the total absence of Tg in the serum of ATC patients and the lack of response of ATC to TSH, a feature which differentiates ATC from PTC. The role of some proteins with still unknown function in thyroid and whose mRNA are greatly downregulated deserves to be investigated: calcyphosine (CAPS) [27], caveolin 2 (CAV2) [28], phosphodiesterases (PDE1A, PDE8B) [7], the potassium channel KCNQ1 gene [29], growth differentiation factors (GDF2, GDF10) and bone morphogenetic proteins (BMP7 (10/11 of ATC), BMP8A).

A downregulation of enzymes involved in H_2_O_2_ metabolism in thyroid (catalase (CAT), metallothioneins (MT1F and MT1G) and superoxide dismutase (SOD3)) was also observed, consonant with the decreased expression of H_2_O_2_ generation genes, DUOXs and DUOXAs [30]. Other genes showing decreased expression encode for transcription factors whose specific role must still be defined: the FOX family (FOXO1, FOXO3, FOXO4, FOXP3), the ID family (ID1, ID3 (10/11), ID4) [31] and Yin Yang 1 (YY1) [32]. Most of these genes were downregulated similarly in PTC and ATC but much more repressed in ATC than in PTC.

#### Fibrosis and calcification

One interesting cluster of Figure 5 is related to bone development, which may be linked to the calcification and the fibrotic processes often observed in ATC [33]; [34]. The last one may at least in part result from inflammation and EMT. With invasion, fibrosis directly leads to the major death threatening strangulation of the patient. Among the genes belonging to this cluster, it is interesting to mention the increased expression of RUNX1, RUNX2, collagen family members (COL1A1, COL5A2 and COL13A1), the bone remodelling cathepsin CTSK, SLC26A2 critical for cartilage and matrix organization, the matrix associated protein SPARC, the osteoblast cadherin CDH11, and the decreased expression of one bone morphogenetic protein (BMP8A).

### ATC Present High Glucose and Glycolysis Metabolism

**Figure 6:**
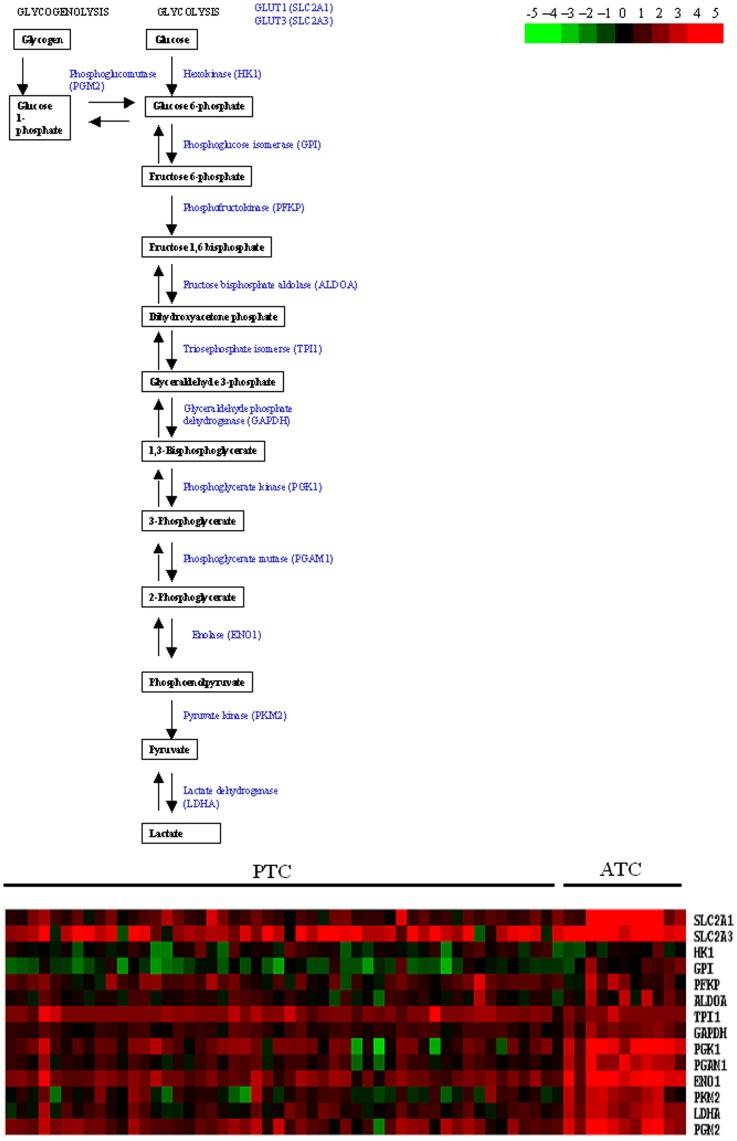
Glycolysis pathway and heatmap with the gene expressions of the intermediate enzymes in ATC and PTC.

Anaplastic carcinomas are often characterized by high fluorodeoxyglucose uptake allowing their visualization by positron emission tomography (PET). This feature illustrates the metabolic shift from an oxidative to a pseudoanaerobic glycolytic metabolism: the Warburg effect. A lot of mRNA encoding enzymes involved in this metabolism were upregulated in ATC (Figure 6). This promotes a high glucose uptake and a pyruvate derivation to lactate production away from mitochondrial metabolism. Similar but weaker effects were observed in PTC. This upregulation of glycolysis is often a consequence of the hypoxic environment which causes the stabilization of the hypoxia inducible factor (HIF1A), which in turn induces anaerobic glycolytic enzymes. While this mechanism probably operates in ATC, it is supplemented by an upregulation of HIF1A mRNA but also of its inhibitor (H1F1AN). It is of interest that hypoxia and HIF induce the upregulation of glycolysis in PTC and ATC [35].

### Angiogenesis in ATC

The expression of angiogenesis factor genes does not give a clear indication of angiogenesis. Indeed, on the one hand, we observed a downregulation of some mRNA encoding angiogenesis factors such as angiopoietin (ANGPTL1) and an upregulation of thrombospondins (THBS1, THBS2) [36] and interferon γ induced proteins (IFI16, IFI30, IFI44) [37], both inhibitors of angiogenesis, but, on the other hand, we observed the upregulation of adrenomedullin (ADM) [38]; [39] and of plasminogen activator and his receptor (PLAU and PLAUR) [40]. All these genes are regulated similarly in ATC and PTC but, again, more strongly in ATC.

### ATC Express Markers of Breast Cancer Stem Cells – Tumor Propagating Cells (CSC-TPC)

A deregulation of the tree main biomarkers [41]; [42] of breast CSC-TPC was observed in our ATC and PTC data i.e. an upregulation of CD44 and ALDH1A3 (10/11 ATC) and a downregulation of CD24.

### Chemoresistance of ATC

The known resistance of ATC to classical chemotherapies could be explained by the presence of a large proportion of CSC-TPC like cells in the tumor and by the increased expression in our data of mRNAs encoding multidrug resistance proteins such as some ATP-binding cassette family members (ABCA8, ABCB10, ABCC5, ABCC10, ABCG1). The acquisition of such tumor resistance mechanisms has also been associated with EMT transition [43].

### The General Role of the Inactivation of the p53 Pathway in ATC is Questioned

The postulated role of p53 inactivation in the important program switch from PTC to ATC [44] may account for some but perhaps not all the transitions. First, only 4 ATC on 11 showed p53 mutation. Second, when analysing by Western Blotting p53 protein expression, an overexpression was found in 4 out of 8 ATC of our study and in 4 out of 8 new ATC but in none of the 7 PTC studied. Moreover, we analysed the protein expression of p53 direct targets, namely mdm2, mdmx, p21 and bax, and no concluding results were obtained (data not shown).

## Discussion

In this study, the molecular phenotypes of ATC and PTC have been correlated with their biological phenotypes. The major shift from normal tissue to ATC and PTC is illustrated by the alteration of more than one third of the genes and by the switches of gene expression within same gene families. This is further multiplied by additional changes in the nature of the mRNA expressed as suggested by the increased expression of mRNA containing the regulatory elements ARE, controlling gene expression.

The downregulation of several immediate early genes (IEG) transcription factors is another major shift and a counter intuitive result. It illustrates, as previously shown by our group [7]; [45], that the upregulation of these genes following growth factor or oncogenic stimulation is not a permanent feature of a stimulated cell but occurs immediately after a signal and before the implementation of a specific program. These upregulations are similar for different signals and programs. This suggests that IEG are necessary to initiate a new program implementation but not to maintain a program [45].

The PTC and FTC origin of the majority of ATC is demonstrated by pathology, mutation analysis and here by gene expression. Pathological examination shows that about 25% of the ATC appear in a differentiated carcinoma (mostly PTC) background [46]; [47]. Mutation of either RAS or BRAF, or PIK3CA and PTEN, which are considered as initial mutations of the differentiated carcinomas are found in the majority of ATC [1]; [48]–[52]. Whereas PIK3CA activation may overlap with RAS and RAF mutations, the latter are generally mutually exclusive. With regard to gene expression, 3 other observations in this work are also compatible with this filiation: first, the large overlap between PTC and ATC gene expression patterns (43% of genes commonly deregulated in PTC are modulated similarly in all the ATC, whereas the other PTC gene deregulations are observed in most ATC and no genes are regulated in an opposite way). Secondly, most of the other regulations in ATC are present in at least some PTC and finally, the number of gene expression changes is greater in ATC than in PTC. The picture of gene regulations in ATC and PTC leaves no doubt about the similarity of these two cancers and about the evolution from the less aggressive PTC to the more aggressive ATC. This does not exclude a FTC origin of some ATC but as FTC are much less frequent than PTC and sometimes misdiagnosed, they were not investigated here. However, all differentiated carcinomas do not evolve in ATC. Indeed, virtually no RET/PTC nor Pax8/PPARγ rearrangements, frequently found in PTC and FTC respectively, have been found so far in ATC [48]; [50]; [51]; [53]. Moreover, the equal male and female prevalence of ATC, as opposed to the female higher frequency of the differentiated carcinomas, suggests also a paradigmatic and non automatic shift [54]. But, whatever the origin of the ATC, deriving from PTC or not, they represent one well defined tumor type characterized by a specific histology, even if the proportion of the different cell subtypes may vary from one tumor to another [55]. The clinical evolution is strikingly similar in all cases [47]: very rapid with the sudden emergence in an enlarged thyroid. The molecular phenotype as demonstrated in this article is also remarkably homogenous between the different ATC tumors originating from different centers.

These different elements suggest that both PTC and ATC exhibit a fundamental program switch compared to the normal tissues. ATC thus results from two major transitions: the first one, a complete gene switch leading to differentiated cancer resulting from the altered expression of ∼ 40% of the genes [10], and the second one, an amplification of this first transition with a few additional features leading to the most aggressive form.

Four candidate mechanisms which may induce a program switch from PTC to ATC have been proposed: the suppression of the p53 pathway, the expression of activated β-catenin and PIK3CA and the acquisition of chromosomal instability. Inactivating mutations of p53 are found in some ATC (36% in our work and >50% in the literature) and overexpression of presumably inactivated p53 protein was detected in an even larger population of one set of ATC (50%) but never in the neighbouring PTC cells [56]. Indirect alteration of p53 function by activation of its inhibitors or inactivation of its activators could account for the other ATC, but our Western Blotting experiments of p53 protein transcription and targets were not conclusive. A general downregulation of this pathway could account in part for the oxidative to glycolytic metabolism switch in this cancer [57]; [58]. ATC with p53 inactivating mutations or inactive protein accumulation did not segregate together in the MDS. Thus, our data are consonant with other studies [59]; [60] and p53 pathway inactivation may account for some but perhaps not all transitions of PTC to ATC. Inactivation of Rb is not a general mechanism: indeed, different ATC derived cell lines display functional Rb proteins [61].

Secondly, activation of β-catenin could represent another mechanism leading to the ATC phenotype. Indeed in one study, such mutations were found in 61% of the lesions but not in the precursor lesions [62]. However β-catenin activating mutations have not been found in this and other studies [63].

Thirdly, the increase in PIK3CA copy number or the presence of activated PI3KCA following mutations, much more prevalent in ATC than in differentiated carcinomas, could also reflect a progression in the cancer phenotype [64]. The increased expression of PI3KCA (8/11) and HIF1 in this study is consonant with this. Activating mutations of ALK, which itself activates both the PI3K and the MAPK cascades have been found in 11.11% of 18 ATC but not in PTC [65].

Finally, some CGH gains and losses specific for ATC have been reported, in addition to common DNA copy number changes described in the precursor differentiated thyroid carcinomas. This may suggest that the development of chromosomal instability underlies tumor progression [64]. Thus at the present time it is difficult to explain the switch from PTC to ATC by a unique mechanism.

The gene expression phenotype of ATC corresponds very well to and explains different clinical and pathological features: hypoxia, glycolysis and fluorodeoxyglucose uptake *in vivo*, EMT and the striking invasive character of the tumors, collagen expression and the fibrotic aspect of the tissue, proliferation markers and the very rapid growth of the tumor, a total loss of differentiation markers and the absence of TSH response and of serum thyroglobulin. The only apparent discrepancy refers to the good vascularization of the tumor which is not reflected by a clear increased expression of angiogenesis markers [19]. On the contrary, gene expression of metabolic enzymes suggests hypoxia. Obviously as in other tumors, vascularization may not correspond to the level of capillary blood flow [66].

When comparing the molecular profiles of PTC and ATC, the two main distinctive gene expression characteristics concern EMT and the repression of differentiation. EMT markers are expressed by both ATC and PTC. However, only ATC present a marked induction of the fundamental transcription factors causing EMT (ZEB, TWIST and SNAIL). This again corresponds very well to the much more invasive phenotype of ATC.

The dedifferentiation phenotype of the ATC cells is well known and is much more severe than the one of the differentiated cancers as attested by the downregulation of thyroid specific differentiation genes and by the total downregulation of the three fundamental thyroid determination factors (TTF1, TTF2 and Pax8): the cells seem to have lost all traces of their thyroid origin. This is also illustrated by the complete absence of expression of Tg and TSHR but also of DUOX1, DUOX2, DUOXA1, DUOXA2, and NKX2-1. These correspond to the *in vivo* biological situation of the ATC which contrary to PTC never respond to TSH stimulation and do not secrete thyroglobulin [67].

As the same tissue displays a high expression of proliferation, EMT and CSC-TPC markers, one could, as is often done, assume that the same cells exhibit these three properties. However, this would run contrary to the often assumed concept that the CSC-TPC would be a slowly proliferating cells [41]; [68]; [69]. Moreover this would contradict the prevalent opinion that EMT cells move but do not proliferate [70].

The fibrosis of ATC is already well known to the pathologist. Whether this results from or induces a strong inflammation is so far unknown. The extensive fibrosis may explain the molecular phenotype of anoxia with the induction of glycolytic enzymes as consequence or as a cause [35]. The latter is in accordance with the fluorodeoxyglucose uptake generally observed in these tumors.

Beside giving a molecular understanding of the pathways involved in tumorigenesis, the identification of proteins corresponding to pathological features allows to propose new putative targets for diagnosis and treatment.

Several characteristics of ATC, demonstrated or confirmed in this work, offer clues about possible therapies to target this so far untreatable disease. The downstream effects of the activated proliferation cascade (eg cell cycle and cell division) would suggest to target common steps of these cascades (i.e. mTOR, cyclin dependent kinases (CDK)) rather than diverse upstream activators. The high proliferation rate should make the tumor sensitive to chemotherapies or radiation therapies, but the low accessibility of the inside of the tumor to blood flow, evidenced by its anoxia, would make it insensitive to these therapy and should be overcome. Therefore pretreatment with capillary blood flow enhancers or treatment in conjunction with high O_2_ supply could improve these classical therapies. Our results also lead to propose other possible therapies: anti-inflammatory treatment to decrease inflammation, a combination of epigenetic and cAMP enhancing treatments to re-establish the differentiation and temporarily the radioiodide uptake, antiglycolytic and antilactate transporter treatments to target the high glycolytic metabolism and, perhaps, the reinduction of expression of the 9 downregulated genes discriminating PTC from ATC. As no single treatment is successful yet, multitargeted approaches should be investigated.

## Supporting Information

Table S1 **Primer sequences, amplicon sizes, targeted exons and PCR conditions (standard condition (Sd): the samples were denaturated at 95°C for 10 min followed by 30 cycles of amplification consisting of 30 sec 95°C, 1 min 60°C ( = Tm) and 1 min 72°C, and a final primer extension of 10 min 72°C.).**
(XLS)

Table S2 **Primer sequences.**
(DOC)

Table S3 **Deregulated mRNA in ATC.**
(XLS)

Table S4 **Deregulated mRNA in PTC.**
(XLS)

Table S5 **GO categories (P-Value <1) corresponding to several biological processes.**
(XLS)
