# Supplementary material for: mRNA Expression in Papillary and Anaplastic Thyroid Carcinoma: Molecular Anatomy of a Killing Switch
Source: PLoS One. 2012 Oct 24;7(10):e37807. doi: 10.1371/journal.pone.0037807 (PMC3480355; doi:10.1371/journal.pone.0037807)
Supplement: Table S2 — Primer sequences. (DOC) [file pone.0037807.s002.doc]

Table S2: primer sequences

NELL2

Fwd CCA GCT GTG AAA CGG ACA TTG A

Rev TCA TGG TAG CCA TCT CTG CAC T

SPINT2

Fwd AAC AGC AAT AAT TAC CTG ACC

Rev AAG GAT GCA CGG CAA GGC

MARVELD2

Fwd TCA GAC AGA TGA TGA GCG AGA

Rev ATG TTC CTG TCG GCT TTC C

DUOXA1

Fwd TGG GTG GAG TCA ACA TCA CAC T

Rev CGT TGT AAT TGA TGG TCT CAT TCA G

RPH3AL

Fwd CGA GGA TCG TCT GCC TTA TT

Rev GCA CGT ACA AGT GTC CAC TAC A

TBX3

Fwd CGA AAT GCC AAA GAG GAT GT

Rev GAA TTC AGT TTC GGG GAA CA

PCYOX1

GCA AGA ATA CGA GGC AGG AG

TGA ACC AGT TGC TCT CCT CA

c5orf41

TTT CGA AGC CAC ACC TTT TC

GCA CCC TCA TTA TCC AGG AC

PKP4

GTG AGC AAG GCA GAC AAC AG

GAG ACA CGC CTG TGC TGA TA
